# The Effect of Adopting New Storage Methods for Extending Product Validity Periods on Manufacturers Expected Inventory Costs

**DOI:** 10.1155/2014/813982

**Published:** 2014-09-11

**Authors:** Po-Yu Chen

**Affiliations:** Department of Advertising, Ming Chuan University, Section 5, 250 Zhong Shan N. Road, Taipei 111, Taiwan

## Abstract

The validness of the expiration dates (validity period) that manufacturers provide on food product labels is a crucial food safety problem. Governments must study how to use their authority by implementing fair awards and punishments to prompt manufacturers into adopting rigorous considerations, such as the effect of adopting new storage methods for extending product validity periods on expected costs. Assuming that a manufacturer sells fresh food or drugs, this manufacturer must respond to current stochastic demands at each unit of time to determine the purchase amount of products for sale. If this decision maker is capable and an opportunity arises, new packaging methods (e.g., aluminum foil packaging, vacuum packaging, high-temperature sterilization after glass packaging, or packaging with various degrees of dryness) or storage methods (i.e., adding desiccants or various antioxidants) can be chosen to extend the validity periods of products. To minimize expected costs, this decision maker must be aware of the processing costs of new storage methods, inventory standards, inventory cycle lengths, and changes in relationships between factors such as stochastic demand functions in a cycle. Based on these changes in relationships, this study established a mathematical model as a basis for discussing the aforementioned topics.

## 1. Introduction

A study by Lobell et al. [1] indicated that, since 1950, corn and wheat yields worldwide have, respectively, declined by 3.8% and 5.5% as the average global temperature has increased. For example, Russian wheat production has encountered a 15% loss. In addition, a study by the Gommes et al. [2] showed that although irrigation technology can effectively counter climate change and increase agricultural production in the initial period, production yields will rapidly decline after 2030 because of factors such as insufficient water resources. Thus, in the next few years, research and development on increasing the income and decreasing the cost of food production will become a crucial topic of discussion. Regarding cost reduction, studies must focus on food storage methods for increasing the effectiveness of food consumption in addition to methods and technologies for reducing postharvest losses. During the rapid development of product storage technologies, perceptions on optimal inventory decisions for fresh merchandise (e.g., fruit and vegetables, flowers, food, medicine, vaccines, and serum) inevitably changed.

Assume that a decision maker who has an inventory of fresh merchandise must confront current (daily) stochastic demands at each time unit (day) and determine the amount of products for sale to minimize the expected total costs in each time unit. These decision problems are similar to the newsboy problem. A typical newsboy problem refers to the product-inventory decision problem regarding a single product at a single time period when stochastic demand occurs. Managers in industries such as fashion, airlines, and hotels all encounter such decision-making situations.

The newsboy problem was initially proposed by Arrow et al. [3] and Morse and Kimball [4]. Since then, considerable extension studies on the concept have been successively proposed. Khouja [5] divided these studies from a single period into 11 detailed categories. Qin et al. [6] continued the study by Khouja [5] and considered the practical conditions of supply chains and generalized extension research and future studies on newsboy problems as three major types: customer demand, supplier pricing policies, and buyer risk profile. Recent studies have focused on the profile of manufacturer risks and considered the decision problems of manufacturers in various risk properties (including risk-averse, risk-neutral, or risk-seeking). Examples of these studies include those by Wang et al. [7], Andersson et al. [8], Wu et al. [9], and Kamburowski [10].

The aforementioned scholars provided elaborate reviews on newsboy problems but did not elaborate on multiperiod or interperiod newsboy problems. Typical newsboy problems assume that products become worthless immediately after expiration. Thus, conditions such as unsold quantity (surplus of goods) and unsatisfied demand (shortage of goods) from the previous period were not included in the inventory decision problem of the coming period. Matsuyama [11] extended Eppen [12] concept of a cross-section newsboy problem, considered time-related research extensions, and proposed the multiperiod newsboy problems model. Matsuyama indicated that if surplus goods existed from the previous period, these goods must be subtracted from the order amounts in the next period; if a shortage of goods occurred during the previous period, this shortage must be considered together with the order amounts in the following period. However, this model still ignored the influence of extended product validity from the previous period on the basis of inventory cost calculations in the next period.

Instead of products, such as newspapers, which have a validity period that is not extendable, the products considered in this study were food and drugs that had validity periods that can be extended. If the inventory decision maker is capable and an opportunity arises, new packaging methods (e.g., aluminum foil packaging, vacuum packaging, high temperature sterilization after glass packaging, or packaging with various degrees of dryness) or storage methods (i.e., adding desiccants or various antioxidants) can be chosen to extend the validity periods of products for several time units (days). Consequently, product purchase decisions that used to be performed at every time unit can instead be performed at every several time units. Under such circumstances, whether manufacturers should adopt new product storage or packaging methods becomes the problem background of this study.

In this paper, a method for the mathematical analysis of advanced calculus, which involves converting between line integral and double integral, also involves converting between multivariate random variables and multiple units random variable. One of the contributions of this research is to apply the mathematical analysis methods for multi-period stochastic demand inventory management.

## 2. Mathematical Model

Assume that an inventory decision maker can choose new product packaging or storage methods to extend the time units of a previous validity period by *k*. In this model, decision makers can select certain numbers from the set {1,2,…, *n*} for *k*. Other relevant symbols are defined as follows.

  *c*(*k*)
: the unit cost (including packaging or storage processing cost) of product validity period for *k* units of time (*k* days);
  (1) $$\begin{array}{c} c\left(k\right)\text{increases}\;\text{as}\;k\;\text{increases},\;k\in \left\{1,2,\ldots ,n\right\} \end{array}$$
 
*c*_*i*_: the per unit time cost for extending the product validity period to *i* period, *i* = 1,2,…, *n*;
 
*Z*_*i*_: the stochastic demand within the *i*th time interval [*i* − 1, *i*], *i* = 1,2,…, *n*;
  
*X*_0_: the stochastic demand within time interval [0, *n*], where
  (2) $$\begin{array}{c} X_{0}=Z_{1}+Z_{2}+\cdots +Z_{n}. \end{array}$$
  When *k* is determined, the stochastic demand *X*_0_ can be decomposed as follows. The stochastic demands (*X*_1_ and *X*_2_) before and after time point *k* are summed; that is, *X*_0_ = *X*_1_ + *X*_2_.
 
*X*_1_: stochastic demand in the previous period;
  (3) $$\begin{array}{c} X_{1}\;\text{is}\;\text{the}\;\text{stochastic}\;\text{demand}\;\text{within}\;\text{time}\;\text{interval}\;\left[0,k\right] \end{array}$$
 
*X*_2_: stochastic demand in the coming period;
  (4) $$\begin{array}{c} X_{2}\;\text{is}\;\text{the}\;\text{stochastic}\;\text{demand}\;\text{within}\;\text{time}\;\text{interval}\;\left[k,n\right] \end{array}$$
 
*E*(*X*_*i*_): expected value of random variable *X*_*i*_, *i* = 0,1, 2;
 
*f*(*x*_1_, *x*_2_): the joint probability density of binary stochastic demand (*X*_1_, *X*_2_) at (*x*_1_, *x*_2_);
 
*f*_*i*_(*x*_*i*_): the probability density of stochastic demand *X*_*i*_ at *x*_*i*_, *i* = 0,1, 2;
  (5) f0(x0)=∫∫x1+x2=x0  f(x1,x2)dx1dx2f1(x1)=∫0∞f(x1,x2)dx2,f2(x2)=∫0∞f(x1,x2)dx1;
 
*F*_*i*_(*x*_*i*_): the accumulated distribution of random variable *X*_*i*_ at *x*_*i*_; that is,
  (6) $$\begin{array}{c} F_{i}\left(x_{i}\right)=\overset{x_{i}}{\underset{0}{\int }}\;f_{i}\left(z\right)dz,\;i=0,1,2; \end{array}$$
 
*h*: the surplus cost of the unit product;
 
*p*: the shortage cost of the unit product.

Model I, Model II, and Model III are presented to describe the effect of extending product validity periods on expected inventory costs. Among these, the mathematical problem of Model III needs, by comparing the results of Model I and Model II, to consider the application of mathematical logic; Model III will be postponed and introduced in (20); Model I is an inventory decision model that comprises previous and coming periods (see (3) and (4)), in which the decision variables are (*s*_1_, *s*_2_); Model II combined the inventory decisions of the previous and coming periods from Model I as a single period inventory decision problem (see (2)) in which the decision variable is *s*_0_.

*s*_1_: the inventory standard determined at time 0 for the stochastic demand distribution *f*_1_ of time interval [0, *k*].
*s*_2_: the inventory standard determined at time *k* for the stochastic demand distribution *f*_2_ of time interval [*k*, *n*].
*s*_0_: the inventory standard determined at time 0 for the stochastic demand distribution *f*_0_ of time interval [0, *n*].

Given the assumption of minimized expected total cost (including purchase cost, holding cost, and shortage cost), the mathematical problems for Model I of the original inventory policy and Model II of the new inventory policy are separately shown in the following:

(7) Model  I:  min⁡(s1,s2)  [π1(s1)+π2(s2)]    =∑i=12  [ci·si+h∫0si(si−z)fi(z)dz        + p∫si∞(z−si)fi(z)dz];

among them the first item in the brackets in (7) represents purchase cost; the second and the third items represent inventory costs; and *c*_1_ = *c*(*k*), *c*_2_ = *c*(*n* − *k*). Consider

(8) Model  II:  min⁡s0  [π0(s0)]    =c0·s0+h∫0s0(s0−z)f0(z)dz     +p∫s0∞(z−s0)f0(z)dz;

among them,

(9) c0=c(n), c0≥c1, c0≥c2  (this  inequality  from  (1)).

## 3. The Optimal Solution of Model

Let (*s*_1_*, *s*_2_*) and *s*_0_* represent the optimal solutions of Model I and Model II, respectively. From (7) and (8), we can obtain

(10) dπ(si)dsi=ci+h∫0sifi(x)dx −p∫si∞fi(x)dx, ∀i=0,1,2=ci+h·Fi(si)−p[1−Fi(si)]=ci−p+(h+p)·Fi(si), ∀si>0

(11) $$\begin{array}{c} \frac{d^{2}\pi \left(s_{i}\right)}{ds_{i}^{2}}=\left(h+p\right)\cdot f_{i}\left(s_{i}\right)>0,\;\forall s_{i}>0 \end{array}$$

(12) 0=dπ(si)dsi|si=si∗=ci−p+(h+p)·Fi(si∗),       that  is  si∗=Fi−1(p−cip+h).

Partially integrating *s*_*i*_ by applying (10) obtains the following:

(13) πi(si)=(ci−p)si+(h+p)∫0si  Fi(z)dz+πi(0)

(14)    =(ci−p)si+(h+p)∫0si  (si−z)  fi(z)dz    +p·E(Xi), i=0,1,2,

where *π*_*i*_(0) = *p* · *E*(*X*_*i*_) can be obtained from (7) and (8).

## 4. A Comparison between Model I and Model II

From (2), (3), and (4), the following is obtained:

(15) $$\begin{array}{c} X_{0}=X_{1}+X_{2},\;\;E\left(X_{0}\right)=E\left(X_{1}\right)+E\left(X_{2}\right). \end{array}$$

**Figure 1:**
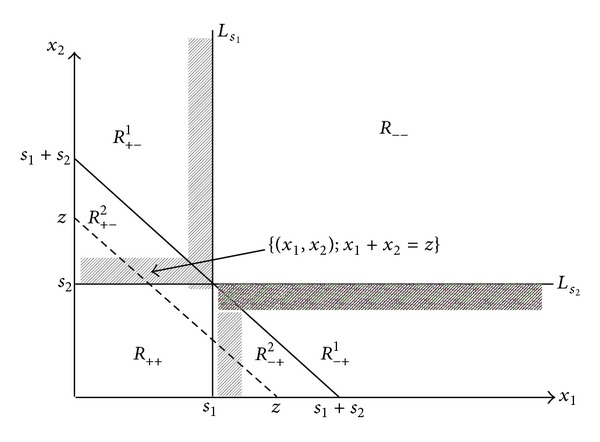
Domains separating function *f*(*x*_1_, *x*_2_) (*s*_1_ and *s*_2_ are given).

This property is substituted into (13) to obtain the following:

(16) [π1(s1)+π2(s2)]−π0(s1+s2) =[(c1−c0)s1+(c2−c0)s2]  +(h+p)[∫0s1(s1−x1)f1(x1)dx1       +∫0s2(s2−x2)f2(x2)dx2         −∫0s1+s2(s1+s2−z)f0(z)dz] =[(c1−c0)s1+(c2−c0)s2]  +(h+p){∫0s1∫0∞(s1−x1)  f(x1,x2)dx2dx1       +∫0s2∫0∞(s2−x2)f(x1,x2)dx1dx2       −∫0s1+s2[∫x1+x2=z(s1+s2−z)‍            ×f(x1,x2)dx1dx2]} =[(c1−c0)s1+(c2−c0)s2]  +(h+p)[∫∫R++∪R+−1∪R+−2(s1−x1)×f(x1,x2)dx1dx2       +∫∫R++∪R−+1∪R−+2(s2−x2)×f(x1,x2)dx1dx2       −∫∫R++∪R+−2∪R−+2(s1+s2−x1−x2)          ×f(x1,x2)dx1dx2](each  region  Rij  is  as  shown  in  Figure  1) =[(c1−c0)s1+(c2−c0)s2]  +(h+p)[∫∫R+−1(s1−x1)f(x1,x2)dx1dx2       +∫∫R−+1(s2−x2)f(x1,x2)dx1dx2       +∫∫R+−2(x2−s2)f(x1,x2)dx1dx2       +∫∫R−+2(x1−s1)f(x1,x2)dx1dx2](this  equation  is  obtained  by  eliminating  the  integrated values  for  each  item  fromthe  previous  equation  in  R++).

Given *s*_1_ and *s*_2_, *R*_+−_ = *R*_+−_^1^ ∪ *R*_+−_^2^ represents the region of surplus goods from the first period (the previous period) and shortage of goods in the second period; *R*_−+_ = *R*_−+_^1^ ∪ *R*_−+_^2^ represents the region of shortage of goods in the first period and surplus goods in the second period.

The four double integrals within the brackets in (16) are all greater than or equal to 0; the meaning of this is presented in order as follows (see Figure 1).

(17) (1)The  average  distance  from    (x1,x2)  to  line  Ls1  when  (x1,x2)∈R+−1.(2)The  average  distance  from    (x1,x2)  to  line  Ls2  when  (x1,x2)∈R−+1.(3)The  average  distance  from    (x1,x2)  to  line  Ls2  when  (x1,x2)∈R+−2.(4)The  average  distance  from    (x1,x2)  to  line  Ls1  when  (x1,x2)∈R−+2.

Equations (13) and (17) showed that the difference in values for optimal solution targets can be represented as follows:

(18) [π1(s1∗)+π2(s2∗)]−π0(s0∗) =[π1(s1∗)+π2(s2∗)−π0(s1∗+s2∗)]  +[π0(s1∗+s2∗)−π0(s0∗)].

Using (16) on the first item and (13) on the second item of the above equation, it yields

(19) [π1(s1∗)+π2(s2∗)]−π0(s0∗) =[(c1−c0)s1∗+(c2−c0)s2∗]+(h+p)  ·{[the  average  distance  from  (x1,x2)  in  (x1,x2)    ∈R+−1∪R−+2  to  line  Ls1∗]   +[the  average  distance  from  (x1,x2)  in  (x1,x2)     ∈R−+1∪R+−2  to  line  Ls2∗]}  +[(c0−p)·|s1∗+s2∗−s0∗|+(h+p)·|∫s1∗+s2∗s0∗  F0(s)ds|],

where *s*_i_* = *F*_*i*_^−1^((*p* − *c*_1_)/(*p* + *h*)), *i* = 0,1, 2 (see (12)).

In (19), only the first item value [(*c*_1_ − *c*_0_)*s*_1_* + (*c*_2_ − *c*_0_)*s*_2_*] is negative and all other items are independent of *c*_1_ and *c*_2_. Thus, if the increased costs of adopting a new packaging or storage method for extending product validity, that is, (*c*_1_ − *c*_0_) and (*c*_2_ − *c*_0_), are relatively smaller than the sum of other inventory costs, then [*π*_1_(*s*_1_*) + *π*_2_(*s*_2_*)] − *π*_0_(*s*_0_*) in (19) has a positive value. In addition, by definition, *s*_0_* (see (8)) is the only minimum of function *π*_0_(*s*_0_). Further discussion regarding the problem of absolute values in (19), which is used to determine whether *s*_0_* or (*s*_1_* + *s*_2_*) is the greater item, is provided in Section 5.

**Figure 2:**
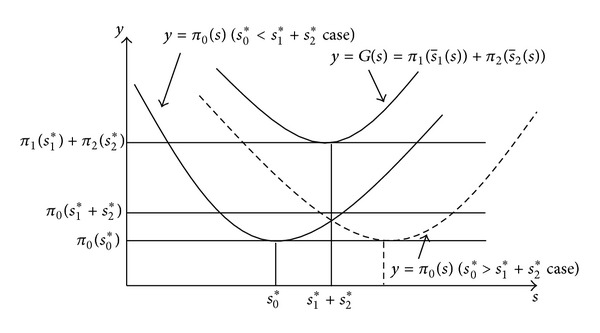
A graphical illustration of (19).

The following proof shows that (*s*_1_* + *s*_2_*) is the only minimum of the objective function *G*(*s*) of Model III (c.f.  Figure 2):

(20) Model  III:  G(s)=min⁡s1,s2⁡[π1(s1)+π2(s2)]

(21)           s.t.    s1+s2=s  (s  is  a  given  parameter).

Let $\left({\bar{s}}_{1}(s),{\bar{s}}_{2}(s)\right)$ be the optimal solution of Model III. Substitute (21) into (20), differentiate *G*(*s*) with respect to *s*_1_, and use (10) and (11) to obtain the following:

(22) dds1[π1(s1)+π2(s−s1)] =(c1−c2)+(h+p)·[F1(s1)−F2(s−s1)] ∀s1d2ds12  [π1(s1)+π2(s2)] =(h+p)·[f1(s1)+f2(s−s1)]>0 ∀s1.

When *s* is given, it is implied that

$\left({\bar{s}}_{1}\left(s\right),{\bar{s}}_{2}\left(s\right)\right)$ is the optimal solution of Model III if and only if

(23) $$\begin{array}{c} \frac{d}{ds_{1}}\left[\pi_{1}\left(s_{1}\right)+\pi_{2}\left(s-s_{1}\right)\right]{|}_{s_{1}={\bar{s}}_{1}(s)}=0,\\ {\bar{s}}_{2}\left(s\right)=s-{\bar{s}}_{1}\left(s\right),\;\forall s. \end{array}$$

Considering differentiation Equation (23) with respect to *s* obtains the following:

(24) 0=dds[F1(s¯1(s))−F2(s¯2(s))]=f1(s¯1(s))·s¯1′(s)−f2(s¯2(s))(1−s¯1′(s))=[f1(s¯1(s))+f2(s¯2(s))]·s¯1′(s)−f2(s¯2(s)).

Thus

(25) s¯1′(s)=f2(s¯2(s))f1(s¯1(s))+f2(s¯2(s))∈[0,1], ∀ss¯2′(s)=1−s¯1′(s)=f1(s¯1(s))f1(s¯1(s))+f2(s¯2(s))∈[0,1], ∀s.

This represents that when s decreases, ${\bar{s}}_{1}(s)$ and ${\bar{s}}_{2}(s)$ also decrease; when *s* increases, ${\bar{s}}_{1}(s)$ and ${\bar{s}}_{2}(s)$ also increase in (25). From (11) and (12), *s*_1_* and *s*_2_* are, respectively, the only minima of *π*_1_(*s*) and *π*_2_(*s*). Equation (25) can be used to obtain the only minimum (*s*_1_* + *s*_2_*) of *G*(*s*). Thus,

(26) G(s1∗+s2∗)=min⁡s  G(s),wherein  s1∗=s¯1(s1∗+s2∗),  s2∗=s¯2(s1∗+s2∗).

A graphical illustration of *G*(*s*) is shown in Figure 2.

## 5. Marginal Effect for Adopting New Product Packaging or Storage Methods

**Figure 3:**
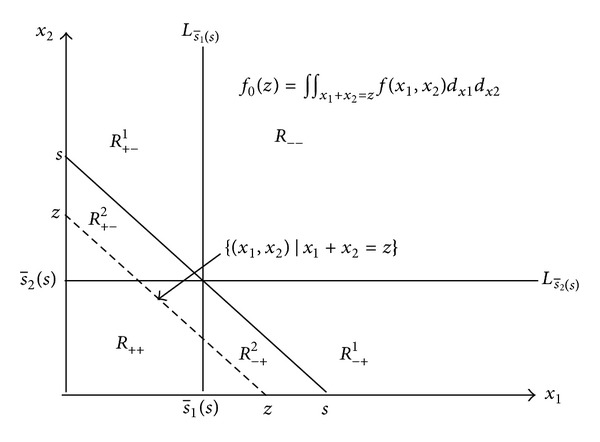
Domains separating function *f*(*x*_1_, *x*_2_) (*s* is given).

Equations (12), (22), and (23) show the following:

(27) dds[π1(s¯(s))+π2(s¯2(s))−π0(s)] =π1′(s¯1(s))·s¯1′(s)+π2′(s¯2(s))·s¯2′(s)−π0′(s) =[(c1−p)+(h+p)·F1(s¯1(s))]·[1−s¯2′(s)]  +[(c2−p)+(h+p)·F2(s¯2(s))]·s¯2′(s)  −[(c0−p)+(h+p)·F0(s)], using  (22) =[(c1−p)+(h+p)·F1(s¯1(s))]  −[(c0−p)+(h+p)·F0(s)] =(c1−c0)+(h+p)  ·[∫∫R++∪R+−1∪R+−2f(x1,x2)dx1dx2   −∫∫R++∪R+−2∪R−+2f(x1,x2)dx1dx2] see  Figure  3 =(c1−c0)+(h+p)  ·[∫∫R+−1f(x1,x2)dx1dx2   −∫∫R−+2f(x1,x2)dx1dx2].

Given the inventory standards s at the beginning of a period, a large (*c*_1_ − *c*_0_) value or a high probability of (*x*_1_, *x*_2_) falling within the *R*_+−_^1^ region or a low probability of (*x*_1_, *x*_2_) falling within the *R*_−+_^2^ region both increase the value in (27), thus increasing the marginal effect of decision makers for adopting new product storage methods. By replacing *s* with (*s*_1_* + *s*_2_*) in (27) and using *G*′(*s*_1_* + *s*_2_*) = 0 (see Figure 2) and (11) and (12), the following property can be obtained:

(28) s1∗+s2∗<s0∗ iff  0<−π0′(s1∗+s2∗) =dds[π1(s¯1(s))+π2(s¯2(s))−π0(s)]|s=s1∗+s2∗ =(c1−c0)+(h+p)·∫∫R+−1f(x1,x2)dx1dx2  −∫∫R−+2f(x1,x2)dx1dx2|s=s1∗+s2∗.

Figure 2 and the previous discussion on marginal effects show that when *s* ∈ (*s*_1_* + *s*_2_*, *s*_0_*), [*G*(*s*) − *π*_0_(*s*)] is reduced as *s* approaches (*s*_1_* + *s*_2_*). This means that if *s*_0_* > (*s*_1_* + *s*_2_*), marginal effect is positive when new product storage methods are adopted. Conversely, if *s*_0_* < (*s*_1_* + *s*_2_*), marginal effect is negative.

## 6. Conclusions

This study provided Models I, II, and III to assist manufacturers encountering inventory decision problems, related to stochastic demands on whether to adopt new product packaging or storage methods for extending product validity and reducing total inventory costs. Model I specifies maintaining the previous conditions of packaging or storage methods; the model consisted of two decision-making periods. The decision maker must individually determine purchase amounts at the beginning of the first and second periods to manage stochastic demands for the periods. In Model II, new product packaging or storage methods are adopted. This allows the combination of the two periods of inventory decision problems in Model I into a single inventory-decision problem period.

A comparison between the total cost [*π*_1_(*s*_1_*) + *π*_2_(*s*_2_*)] of the optimal solution (*s*_1_*, *s*_2_*) of Model I and the total cost *π*_0_(*s*_0_*) of the optimal solution *s*_0_* of Model II showed the following.

1. Although the single period inventory-decision approach of Model II supports higher purchase costs more than the two-period approach of Model I (see the first item of (19)), comparatively lower inventory costs had to be paid (see the second and third items of (19)).
2. A reduced probability of surplus goods in the first period and of shortage of goods in the second period (i.e., the (*x*_1_, *x*_2_) ∈ *R*_+−_ region in Figure 1) or of shortage of goods in the first period and of surplus of goods in the second period (the (*x*_1_, *x*_2_) ∈ *R*_−+_ region in Figure 1) minimizes the effect discussed in (1).
  - In special conditions, when the correlation coefficients of demand from the first period *x*_1_ and those from the second period approached 1 (in contrast to −1), the chance of (*x*_1_, *x*_2_) ∈ *R*_++_ ∪ *R*_−+_ is reduced instead of being increased. Accordingly, the aforementioned effect is also reduced.
  - In special conditions, when demands from the first and second period (*x*_1_, *x*_2_) within *R*_−+_ ∪ *R*_++_ are concentrated in the proximity of lines *L*_*s*_1__* and *L*_*s*_2__* (the diagonally shaded region in Figure 1), the aforementioned effect increased.
3. When the probability of demands from the first and second period (*x*_1_, *x*_2_) falling within *R*_+−_^1^ as compared to falling within *R*_−+_^2^ increased, this supported the optimal inventory standard *s*_0_* of Model II being larger than the optimal inventory standard (*s*_1_* + *s*_2_*) of Model I (see (28)).

The aforementioned results were specific to the optimal solutions and target values for Models I and II. Regarding inventory standards s for any nonoptimal solution, this study also included a discussion on the marginal effect of adopting new product storage methods at point *s* (shown in (27), Figure 3, and Model III).
